# Lewy Body Variant of Alzheimer's Disease: Selective Neocortical Loss of t-SNARE Proteins and Loss of MAP2 and α-Synuclein in Medial Temporal Lobe

**DOI:** 10.1100/tsw.2009.151

**Published:** 2009-12-16

**Authors:** Elizabeta B. Mukaetova-Ladinska, John H. Xuereb, Francisco Garcia-Sierra, Jenny Hurt, Herman-J. Gertz, Richard Hills, Carol Brayne, Felicia A. Huppert, Eugene S. Paykel, Magnus A. McGee, Ross Jakes, William G. Honer, Charles R. Harrington, Claude M. Wischik, CC75C Collaboration Group

**Affiliations:** ^1^ Institute for Ageing and Health, Newcastle University, United Kingdom; ^2^ Department of Pathology, University of Cambridge, United Kingdom; ^3^ Cambridge Brain Bank Laboratory, MRC Centre, Cambridge, UK; ^4^ Department of Cell Biology, CINVESTAV - I.P.N., Mexico City, Mexico; ^5^ Department of Psychiatry, University of Leipzig, Germany; ^6^ Department of Public Health, University of Cambridge, UK; ^7^ Department of Psychiatry, University of Cambridge, UK; ^8^ Department of Public Health and General Practice, Christchurch School of Medicine and Health Sciences, University of Otago, Dunedin, New Zealand; ^9^ Laboratory of Molecular Biology, MRC Centre, Cambridge, UK; ^10^ Department of Psychiatry, University of British Columbia, Vancouver, BC, Canada; ^11^ Division of Applied Health Sciences, University of Aberdeen, UK

**Keywords:** Alzheimer’s disease, dementia with Lewy bodies, tau, MAP2, t-SNARE complex, α-synuclein

## Abstract

Lewy bodies (LBs) appear in the brains of nondemented individuals and also occur in a range of neurodegenerative disorders, such as dementia with Lewy bodies (DLB) and Parkinson's disease. A number of people with a definite diagnosis of Alzheimer's disease (AD) also exhibit these intraneuronal inclusions in allo- and/or neocortical areas. The latter, referred to as Lewy body variant of AD (LBV), bears a clinical resemblance to AD in terms of age at onset, duration of illness, cognitive impairment, and illness severity. Since the presence of LBs is accompanied by neuronal cytoskeleton changes, it is possible that the latter may influence neuronal connectivity via alterations to the synaptic network. To address this, we examined the expression of synaptic proteins (synaptophysin, syntaxin, SNAP-25, and α-synuclein) and two cytoskeletal proteins (tau and MAP2) in the brain tissue of subjects enrolled in a population-based autopsy study (n = 47). They were divided into groups with no memory problems (control group, n = 15), LBV (n = 5), AD devoid of LBs (n = 17), cerebrovascular dementia (n = 3), and mixed dementia (n = 7). The LBV and AD groups had a similar degree of cognitive impairment and neuropathological staging in terms of Braak staging and CERAD score. In comparison with the control group and the dementia groups without LBs, the LBV group had significantly lower levels of syntaxin and SNAP-25 (23%) in the neocortex, and depletion of MAP2 (64%), SNAP-25 (34%), and α-synuclein (44%) proteins in the medial temporal lobes. These findings suggest that the t-SNARE complex deficit present in LBV may be associated with the presence of LB-related pathology and may explain the more profound cholinergic loss seen in these patients.

## INTRODUCTION

Cytoplasmic Lewy bodies (LBs) occur in a range of neurodegenerative disorders associated with dopaminergic neuronal degeneration, including Parkinson's disease and dementia with Lewy bodies (DLB). However, LBs are seen in smaller numbers in older people with no cognitive impairment[1] and in other neurodegenerative disorders, including Down syndrome and Alzheimer's disease (AD). One- to two-thirds of people with a definite diagnosis of AD will have LBs in their allo- and/or neocortical areas[2,3,4], and these define the Lewy body variant of AD (LBV) or DLB with AD[5,6]. In a recent neuropathological survey, the LBV was confirmed in 18% of the autopsy material[7].

Both AD and LBV appear to share similar genetic risk factors (including family history of dementia or APOE ε4 allele frequency)[8], as well as clinical characteristics and course (e.g., age at onset, duration, extent of cognitive impairment, or performance on activities of daily living)[8,9]. However, differences in LBV clinical presentation have been described in terms of sensitivity towards neuroleptic medication, more frequent parkinsonian features, greater incidence of delusional and hallucinatory experiences, and more profound impairment in visuospatial and executive tasks, as well as cognition[10,11,12,13,14,15]. Despite a majority of studies that report a similar extent of cognitive decline and mortality rate between AD and LBV, faster cognitive decline and accelerated mortality in LBV have also been described[16,17].

Although some similarities between AD and LBV have been found in a correlative cliniconeuropathological study[18], the majority of the cliniconeuropathological and biochemical studies have reported conflicting results on the influence of both neurofibrillary pathology and synaptic protein loss, and on the effect of LB pathology on the extent of cognitive impairment and decline in LBV[15,19,20]. Thus, the presence of LBs may be a major correlate of cognitive decline in both DLB and LBV[21,22], although not necessarily with the overall clinical presentation or disease duration[23,24].

Since the presence of LBs is accompanied by alterations to the neuronal cytoskeleton[25] and has an impact on the regulation of synaptic function[26], there is a possibility that LBs may contribute to more extensive alterations to the neuronal network, and thus be closely related to the clinical presentation and cognitive decline in LBV subjects. This might include more generalized synaptic loss as a result of both neurofibrillary and LB-related neurodegeneration. To investigate this further, we have undertaken an extensive immunobiochemical study on a prospectively clinically assessed cohort. Despite having a similar degree of cognitive impairment, Braak stage, CERAD scoring, and accumulation of phosphorylated tau protein to individuals with a definite diagnosis of AD, the LBV group showed a selective t-SNARE (target soluble N-ethylmaleimide-sensitive factor attachment protein receptor protein) complex loss in the neocortical areas of frontal and temporal lobes in relation to AD subjects. The LBV group also had lower levels of SNAP-25, MAP2, and α-synuclein proteins in the medial temporal lobe. These findings suggest that the co-occurrence of LB and AD pathology in LBV contributes to the lowering of synaptic protein expression in both neo- and allocortical areas, alters the allocortical dendritic network, and may underlie the profound cholinergic deficit in the LBV subjects.

## MATERIALS AND METHODS

### Case Material

The brain tissue used for our study came from 47 subjects (32 women and 15 men) in the Cambridge Project for Later Life (CPLL[27,28]). In addition to undergoing prospective clinical and neuropsychological assessments, these individuals agreed to donate their brain tissue for research purposes[29]. They have been fully characterized in earlier neuropathological and biochemical studies (Table 1)[30,31]. The study consisted of five groups: (1) 15 individuals having no cognitive impairment (control group); (2) three with a definite diagnosis of cerebrovascular dementia (CVD), without AD[32]; (3) 17 AD; (4) seven with mixed AD and CVD dementia (mixed dementia group[32]); and (5) five in which AD is associated with LBs in either the brainstem and midbrain (n = 2) and/or neocortical (n = 3) areas (LBV).

**TABLE 1**
**Clinical and Neuropathological Characteristics of Groups***

| Groups | N | Age at Death (Year) | Clinical Severity | Braak Stages | CERAD Rating** |
|---|---|---|---|---|---|
| Control | 15 | 88.8 ± 0.9 | 0.3 ± 0.3 | 2.7 ± 0.2<br>(I-IV) | 0 (n = 13)<br>s (n = 4) |
| CVD | 3 | 90.7 ± 5.7 | 3.7 ± 0.4 | 2.7 ± 0.3<br>(II-III) | 0 (n = 2)<br>s (n = 1) |
| AD | 17 | 88.8 ± 1.1 | 2.1 ± 0.3 | 4.3 ± 0.9<br>(III-VI) | 0 (n = 3)<br>s (n = 6)<br>m (n = 5)<br>f (n = 3) |
| Mixed | 7 | 87.7 ± 1.0 | 2.4 ± 0.2 | 0.4 ± 0.6<br>(I-V) | 0 (n = 2)<br>s (n = 2)<br>m (n = 3) |
| LBV | 5 | 88.0 ± 1.9 | 3.0 ± 0.5 | 3.4 ± 0.7<br>(III-V) | 0 (n = 1)<br>s (n = 1)<br>m (n = 3) |
| ANOVA | | F = 0.32; *p* = 0.865 | F = 12.56; *p* = 0.0001 | F = 4.43; *p* = 0.004 | F = 4.67; *p* = 0.003 |

* The rating of the clinical severity of cognitive impairment was done according to the 5-point scale of CAMDEX.

** CERAD protocol (presence of plaques): s = seldom, m = moderate, f = frequent.

The subjects, all over the age of 75 years at the beginning of the study, were followed prospectively up to three times: at 2.4, 6.0, and 10.0 years after the study began. The severity of cognitive impairment (graded on a 5-point scale) was rated using the CAMDEX diagnostic schedule, as previously reported[31]. Clinical status during the 12 months prior to death was determined either by direct assessment or by retrospective informant interview. None of the patients with LB pathology (LBV group) had clinical features typical of DLB. The average age at death was 88.7 ± 0.6 years (mean ± SE), with no difference between the age at death for the female and male subjects. Similarly, the age at death did not differentiate among the groups (F = 0.32, $p$ = 0.865; Table 1).

## Tissue Sampling, Morphometric Analysis, and Braak Stage

At the time of limited autopsy, one cerebral hemisphere and the contralateral cerebellar hemisphere were fixed, and the remaining hemisphere was sliced into 1-cm-thick slices in the coronal plane and snap frozen. The brain sampling was performed on these frozen sections, taking the grey matter of the association areas of the frontal (BA9 and 10) and temporal (BA 21 and 22) neocortical areas, and the medial temporal lobe, subdissected into hippocampus (containing Ammon's horn and subiculum) and entorhinal cortex (BA38). Uniformity of sampling was maintained by using internal neuroanatomical markers when taking tissue from the frozen slices, i.e., head of caudate and the beginning of ventricles

(for the frontal areas), and the junction between the amygdala and hippocampus (for temporal and medial temporal lobe areas).

For morphometry and staging procedure, a previously published protocol was used[30]. Briefly, paraffin-embedded sections from formalin-fixed tissue (7 µm thickness) were cut from 11 brain areas and stained with hematoxylin and eosin (to assess nerve cell loss, gliosis, and ischemic change), congo red (vascular amyloid deposits), mAb 11.57 (neurofibrillary tangles and neuritic plaques), amyloid (Aβ) immunohistochemistry (using an immunoprobe donated by Dr. M. Landon, Department of Biochemistry, University of Nottingham), and an antiubiquitin polyclonal serum pAb BR251 (LBs). Neuropathological staging was performed by two of us (JX and HG) according to the criteria of Braak and Braak[33] and the CERAD protocol[34]. The former gives a measure of neurofibrillary pathology, the latter of amyloid pathology. The morphometric analyses have been described previously[30].

The AD and LBV groups were Braak stage III or greater. As expected, the AD group showed significantly greater neurofibrillary pathology in relation to the control and CVD groups (t = 4.77, $p$ = 0.0001 and t = 2.63, $p$ = 0.017, respectively; Table 1). Plaque distribution, as assessed according to the CERAD protocol, discriminated between the groups: the control subjects and those with CVD had a substantially lower CERAD stage than the AD, LBV, and mixed dementia groups. However, the latter three groups exhibited similar plaque distribution (Table 1).

## Biochemical Analysis and Enzyme-Linked Immunoassays (ELISA)

In all experiments, 0.3–0.5 g of frozen brain tissue was used. The brain tissue was homogenized in 0.32 *M* sucrose, and the homogenate divided into two equal portions. One portion was processed through an A68 protocol containing the synaptosomal preparation, and the other through an if-II protocol, containing heat-stable microtubule-associated proteins and α-synuclein, as described[35].

### *Phosphorylated Tau and Synaptosomes*

Following homogenization, an equal volume of 1 *M* NaCl was added to the brain homogenate and this material was centrifuged at 13,000 *g* for 15 min. The supernatant was used for preparation of synaptosomes (containing synaptophysin, syntaxin, and SNAP-25)[31]. The pellet was rehomogenized in 0.32 *M* sucrose, and an equal volume of buffer containing 2 *M* NaCl, 1 m*M* $MgCl_2$, 2 m*M* EGTA, 0.32 *M* sucrose, and 200 m*M* MES (pH 6.5) was added, followed by centrifugation in a Beckman TL100 ultracentrifuge at 25,000 *g* for 15 min at 4°C. The supernatant was removed and sarkosyl was added to a final concentration of 1%; the mixture was incubated with gentle rotation at 25°C for 1 h. It was then centrifuged at 200,000 *g* for 30 min at 4°C. The supernatant was discarded and the pellet (A68) was retained for analysis of sarkosyl-insoluble phosphorylated tau protein using mAb AT8.

### *MAP2 and α-Synuclein*

The brain homogenate was centrifuged at 85,000 *g* for 15 min. The supernatant was used to prepare an S1 fraction containing heat-stable proteins as described previously[31]. The pellets, containing MAP2 and soluble α-synuclein, were suspended in 500 µl $NH_4HCO_3$ (50 m*M*, pH 8.0) and analyzed for MAP2 (mAb C) and α-synuclein (pAb PER2).

### *Immunoassays*

The measurement of phosphorylated tau protein in the A68 preparation was estimated with a competitive ELISA using mAb AT8, which recognizes pSer202/pThr205 of tau protein. The levels of synaptophysin, syntaxin, and SNAP-25 were determined using an indirect ELISA, with mAbs EP10 (1:10), SP8 (1:100), and SP12 (1:100), respectively. The characteristics of these antibodies have been described previously[36]. The immunohistochemical profile of these immunoprobes corresponds to their immunobiochemically detected protein expression (Fig. 1)[37]. The level of α-synuclein was also determined in an indirect ELISA, using a polyclonal serum PER2 (1:2000) raised against a C-terminal region of the α-synuclein molecule, and its characteristics have been described previously[38].

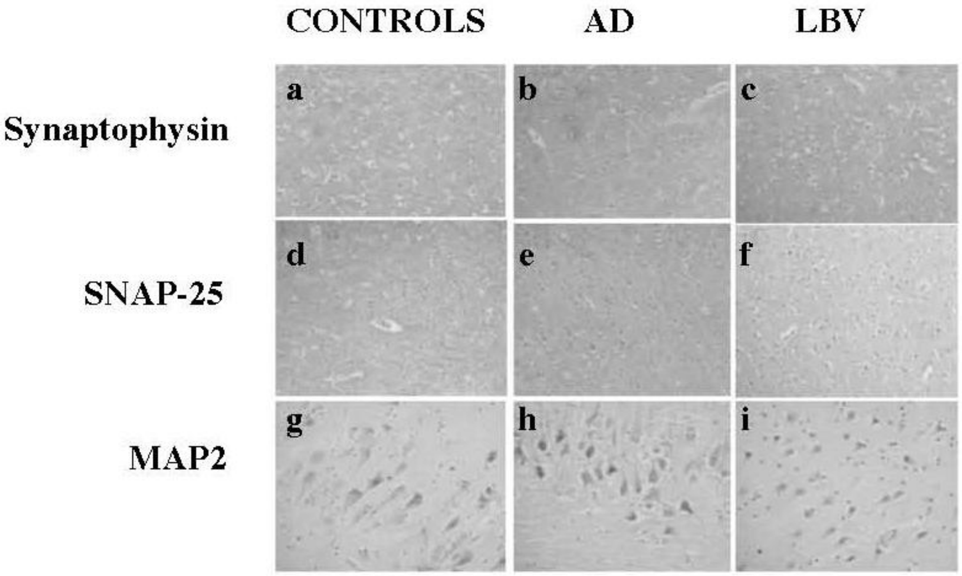

**FIGURE 1.** Immunohistochemical profile of synaptic and MAP2 immunoprobes in aging, AD, and LBV. Synaptophysin immunostaining was similar between the control (a), AD (b), and LBV (c) groups, whereas the LBV subjects showed significant reduction in SNAP-25 (f) in relation to the control (d) and AD (e) subjects. In the hippocampus, the extent of intraneuronal MAP2 accumulation and dendritic arborization in the hippocampus was lower in the AD group (h), with much more profound loss present in LBV subjects (i) in comparison to the control group (g). Immunohistochemistry: synaptophysin (mAb EP10, 1:10; a–c), SNAP-25 (SP12, 1:10; d–f), MAP2 (mAbHM2, Sigma, Poole, U.K., 1:100; g–i). Frontal cortex, a–f; hippocampus, g–i. Magnification ×100 (a–f) and ×400 (g–i).

The level of MAP2 protein was determined using a competitive ELISA, with mAb C (1:2000) and a synthetic peptide corresponding to MAP2c sequence (50 ng/ml) serving as a solid phase (gift from Dr. A. Matus, Basle). Each sample was analyzed in triplicate at six dilutions, and assay curves were plotted using Softmax, version 2.0 (Molecular Devices Corp). All values have been normalized for 0.3-ml fraction from 0.3–0.5 g of brain tissue and expressed as relative units of immunoreactivity.

## Statistical Analysis

Measurements of phosphorylated tau protein, synaptic protein markers, and MAP2 were all log-transformed prior to analysis [value = ln (value +1)]. For the purpose of analysis, neocortical (frontal and temporal association areas) and medial temporal lobe (hippocampus and entorhinal cortex) areas were analyzed separately. This was done partly because of the small number of cases in individual groups, and also because there was no major significant regional difference in the expression of phosphorylated tau, synaptic, and MAP2 proteins in the two distinct neocortical or medial temporal lobes when analyzed separately (data not shown). We have used ANOVA analysis to determine differences in the protein levels among different diagnostic groups, as well as to address the differences in the protein expression patterns between two samples/groups. Values for mean difference, standard error of the mean, and *p* values are presented. The analyses were undertaken using SPSSv15 for Windows.

## RESULTS

### Phosphorylated Tau Protein

As expected, there were higher levels of phosphorylated tau in the medial temporal lobe compared with neocortical areas for all controls, CVD, AD, and mixed dementia (M) subjects ($p = 0.0001$, $p = 0.008$, $p = 0.0001$, and $p = 0.006$, respectively) compared to the neocortical areas. Surprisingly, this was not the case for the LBV subjects in whom the level of phosphorylated tau was similar for both the neocortical and medial temporal lobe areas ($p = 0.125$; Fig. 2a and b).

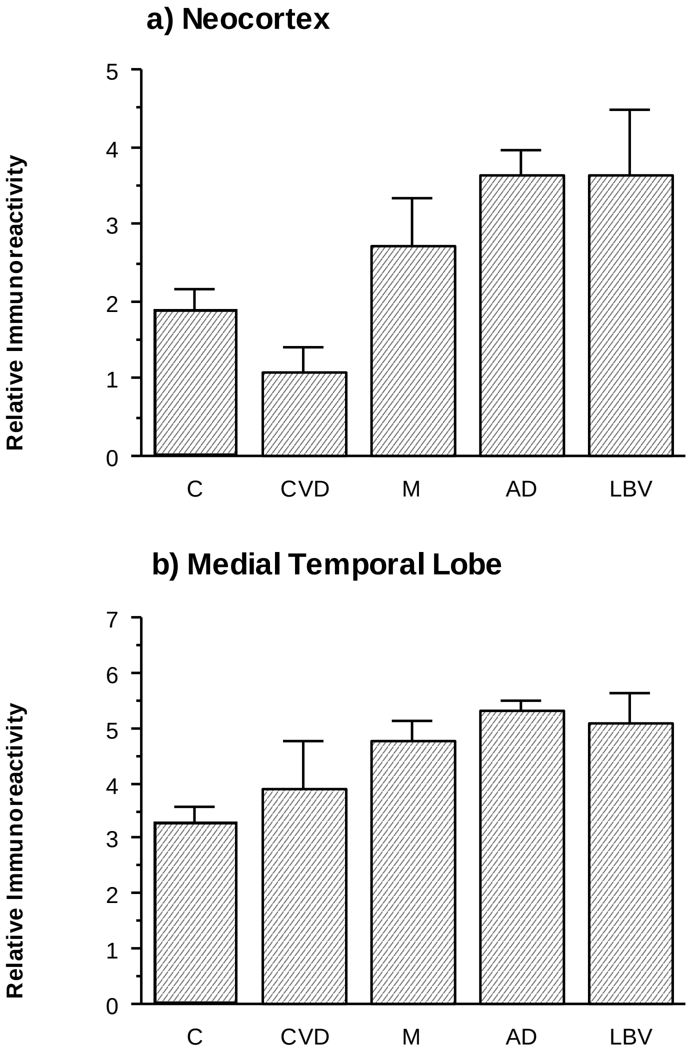

**FIGURE 2.** Distribution of phosphorylated tau protein. The level of phosphorylated tau protein, measured in relative immunoreactivity units, differed significantly among the groups (ANOVA analysis: $F = 2.72$, $p = 0.035$ for neocortical areas, and $F = 3.33$, $p = 0.014$ for medial temporal lobe).

For medial temporal lobe, the highest level of phosphorylated tau protein was found in the AD and LBV groups ($p$ = 0.0001 and $p$ = 0.004, respectively, compared with the control group), followed by the mixed dementia group ($p$ = 0.003 in relation to the control group; Fig. 2b). The level in AD was 38% greater than in the age-matched controls. There was no difference in the level of phosphorylated tau between these three groups (AD vs. LBV, $p$ = 0.782; AD vs. M, $p$ = 0.149; LBV vs. M, $p$ = 0.653), and between the CVD and control groups ($p$ = 0.411). Although the mixed dementia group had a 17% higher level of phosphorylated tau, this did not reach statistical significance when compared to that of the CVD group ($p$ = 0.238).

The neocortical measures of phosphorylated tau showed a significant difference among the analyzed control and dementias groups (F = 5.60, $p$ = 0.0001, Fig. 2a). Thus, the AD and LBV groups had significantly (47%) elevated levels of phosphorylated tau protein in the temporal and frontal neocortices ($p$ = 0.0001 and $p$ = 0.007 for AD and LBV groups, respectively, compared to control subjects; Fig. 2a). Similarly these two groups had threefold higher levels of neocortical phosphorylated tau protein in relation to the elderly group with a definite diagnosis of CVD ($p$ = 0.002 and $p$ = 0.030 for AD and LBV, respectively). The neocortical measurements of phosphorylated tau protein did not discriminate between the AD and the LBV groups ($p$ = 0.995), or the mixed dementia group ($p$ = 0.134 and $p$ = 0.343 for AD and LBV, respectively). The control groups had similar levels of phosphorylated tau protein as the CVD and the mixed dementia groups ($p$ = 0.135 and $p$ = 0.145, respectively).

## Distribution of Synaptic Proteins

### *Synaptophysin*

The distribution of synaptophysin in neocortical areas indicated that all groups, with the exception of LBV subjects, had similar levels of this synaptic protein (F = 0.99, $p$ = 0.418; Fig. 3a). The LBV group had 10% less synaptophysin in the neocortical areas of the frontal and temporal lobes in relation to the control group ($p$ = 0.057; Fig. 3a). In contrast, the medial temporal lobe expression of synaptophysin was similar irrespective of diagnosis (F = 0.56, $p$ = 0.695; Fig. 3b).

### *Syntaxin*

There were significant differences among the groups in the level of neocortical expression of syntaxin (F = 3.15, $p$ = 0.018; Fig. 3a) and this was largely due to the lower level of syntaxin in the LBV group. Syntaxin was significantly depleted in the LBV neocortex when compared with the control (27%; $p$ = 0.0001), AD (25%, $p$ = 0.004), and mixed dementia groups (23%, $p$ = 0.025) (Fig. 3a), but similar to that detected in CVD subjects ($p$ = 0.118). In contrast, the expression of syntaxin in the medial temporal lobe did not differ between groups (F = 1.12, $p$ = 0.350; Fig. 3b).

### *SNAP-25*

The distribution of SNAP-25 was similar to that of syntaxin, showing significant neocortical depletion in the LBV group in relation to normal aging (27%, $p$ = 0.0001), AD (20%, $p$ = 0.013), and mixed dementia (17%, $p$ = 0.063) (Fig. 3a). The mixed form of dementia also had a tendency to have less SNAP-25 in the neocortical areas of the frontal and temporal lobes in relation to the control group (11%, $p$ = 0.072; Fig. 3a). Similarly, there was a significant depletion of SNAP-25 in the medial temporal lobe in all dementia groups, irrespective of the underlying neuropathology. Thus, the AD, LBV, CVD, and mixed dementia groups had significantly less SNAP-25 in their medial temporal lobes compared with controls (up to 38%, $p$ = 0.0001; Fig. 3b). However, LBV and CVD cases had similar levels of SNAP-25, and both groups had significantly less SNAP-25 than the AD group (21–26%, respectively; $p$ = 0.0001), despite the presence of greater neuropathology in the latter (Table 1).

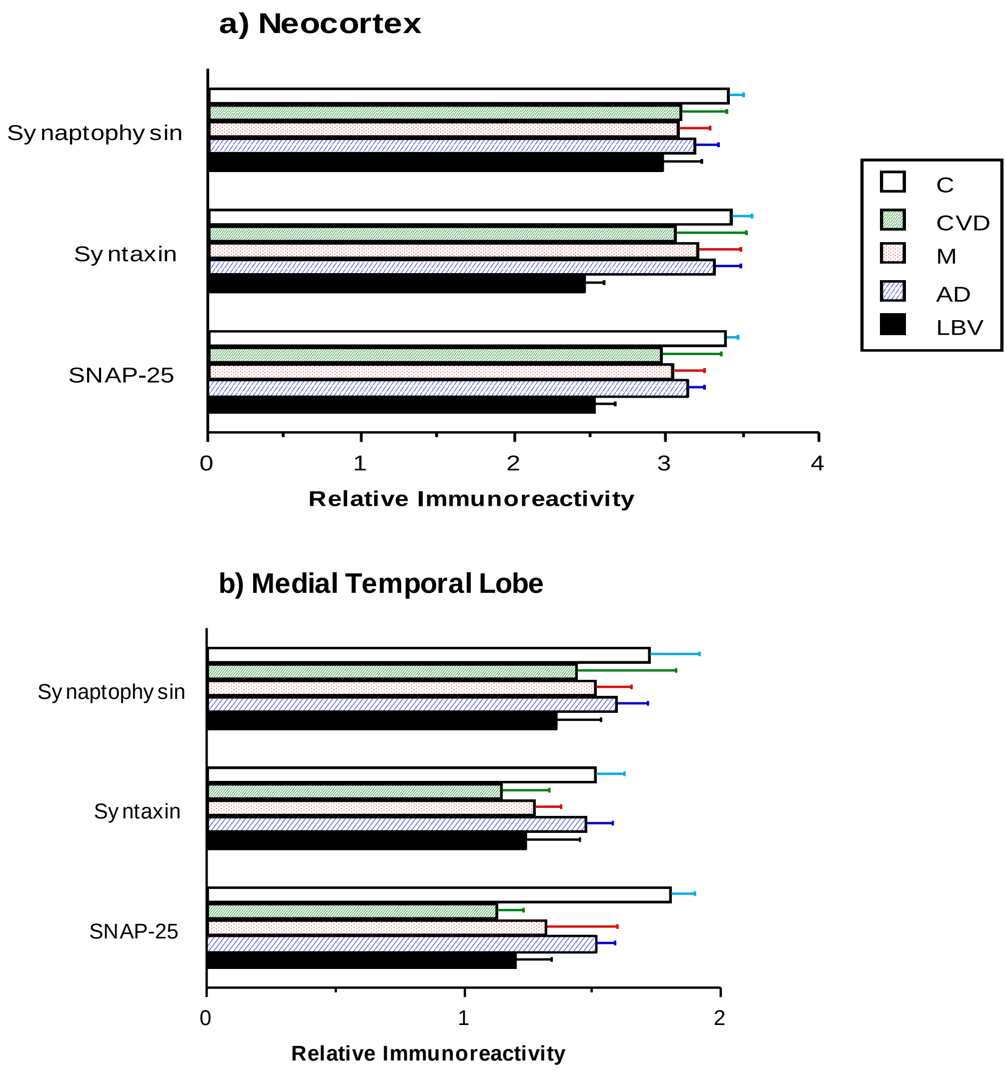

**FIGURE 3.** Distribution of synaptic proteins. The levels of synaptophysin were similar in the groups for both (a) neocortical (F = 0.99, $p$ = 0.418) and (b) medial temporal lobe areas (F = 0.56, $p$ = 0.695). The syntaxin levels were different within the groups in the neocortical areas (F = 3.15, $p$ = 0.018), but not in the medial temporal lobe (F = 1.12, $p$ = 0.350). SNAP-25 levels differed significantly between the groups for both the neocortical (F = 3.69, $p$ = 0.008) and medial temporal lobe areas (F = 6.55, $p$ = 0.0001).

## MAP2 and α-Synuclein Distribution

The neocortical MAP2 levels were largely unaffected by the neurodegenerative process (F = 1.37, $p$ = 0.252; Fig. 4a). In contrast, the medial temporal lobe expression of MAP2 was somewhat different (F = 5.28, $p$ = 0.001). Thus, the LBV cases had substantially less MAP2 (52–55%), discriminating them from the AD ($p$ = 0.001) and control ($p$ = 0.001) groups, respectively (Fig. 4b).

Interestingly, the neocortical expression of α-synuclein was similar between all groups, with the exception of the mixed form of dementia, which had a 30% lower level compared to the control group ($p$ = 0.034) and all other forms of dementia (CVD: 38%, $p$ = 0.034; AD: 22%, $p$ = 0.010; and LBV: 37%, $p$ = 0.044; Fig. 4a). In contrast, the LBV group had the least α-synuclein in the medial temporal lobe (46–56%), significantly discriminating this group from the control ($p$ = 0.022), AD ($p$ = 0.003), and mixed dementia groups ($p$ = 0.001) (Fig. 4b), but similar to that of the vascular dementia group.

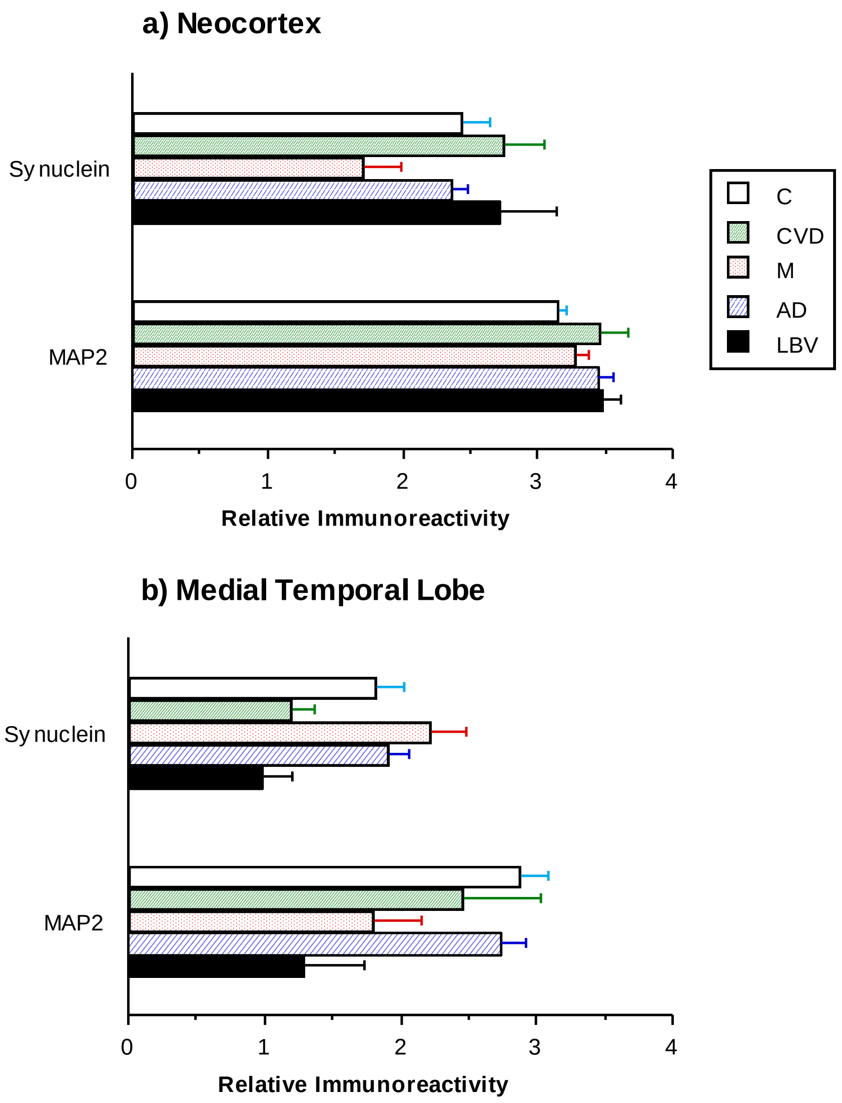

**FIGURE 4.** Distribution of α-synuclein and MAP2. The levels of α-synuclein and MAP2 protein were significantly different among the groups for the medial temporal lobe (b; F = 3.43, $p$ = 0.012 and F = 5.28, $p$ = 0.001, respectively), whereas the level of these proteins did not discriminate among the groups in the neocortical areas of the frontal and temporal lobes (a; F = 2.45, $p$ = 0.052 and F = 1.37, $p$ = 0.252, for α-synuclein and MAP2, respectively).

## DISCUSSION

In this study, we demonstrate that individuals with LBV have substantially lower levels of t-SNARE complex synaptic proteins in the frontal and temporal neocortices, as well as SNAP-25, MAP2, and α-synuclein proteins in their medial temporal lobes compared to control and AD subjects. The more profound and generalized loss of these proteins suggests that the combined presence of AD and LB-related pathology has a profound effect on both the synaptic and dendritic network in LBV.

LBV is generally regarded as a subgroup of AD lacking neurofibrillary tangles, and one in which the Braak pathological stage is lower at the time of death[5,21]. In our study, both AD and LBV groups were of similar Braak stage, a feature also reflected in similar levels of phosphorylated tau protein. This excludes the possibility that the observed decrease in synaptic proteins in LBV arises from differences in the extent of neurofibrillary pathology seen in LBV and AD. This contrasts with an immunohistochemical study in which hippocampal synaptophysin loss in DLB was related to Braak stage and neuritic degeneration[39]. Furthermore, in our previous studies, we demonstrated that the observed differences in the expression of synaptic and cytoskeletal proteins in aging and dementia cannot be attributed or reflect age at death, duration of disease process, and postmortem delay[40,41].

Loss of certain synaptic proteins, especially synaptophysin, has been demonstrated in LBV. Thus, substantial depletion of synaptophysin in the frontal neocortex[42,43] appears to be equal to that observed in AD[19,22,43,44]. This has been confirmed in our study. Furthermore, SNAP-25 loss in medial temporal lobes was observed in all dementia groups. This would suggest that the selective medial temporal lobe loss of SNAP-25 may be associated with the clinical dementia syndrome, and may also reflect the underlying associated neurotransmitter deficits.

In addition to α-synuclein, a range of proteins are associated with LBs, including synaptophysin, amyloid precursor protein[45], and cytoskeletal proteins, such as MAP2[46] and neurofilament[26]. The more profound synaptic loss in LBV in relation to the AD group may be attributable, therefore, to the allo- and/or neocortical presence of LBs, the LB influence upon expression of synaptic proteins (especially those involved in docking and fusion of synaptic vesicles, such as syntaxin and SNAP-25), or, alternatively, to differences with respect to incorporation of synaptic proteins within neuritic plaques. However, this is not supported by our findings that there is a low density of LBs in both allo- and neocortical areas, and the similar extent to which neuritic plaques are observed in LBV and AD groups (Mukaetova-Ladinska et al., unpublished data) rule out this possibility.

Although microtubule-associated proteins such as MAP1 and MAP5 have been described as an integral part of LBs, this is the first study to address the distribution of MAP2 protein in LBV. Two studies have reported the presence of MAP2 immunostaining in LBs. A limited number of LBs in the brainstem and sympathetic ganglia in Parkinson's disease appear to be positive for MAP2[47], with MAP2 colocalizing with α-synuclein and ubiquitin in LBs and neuronal nuclei[46]. MAP2 colocalization with α-synuclein in LBs may provide a further explanation for the extensive loss of both these proteins within the same brain regions in subjects with LBV.

α-Synuclein has been implicated in the pathogenesis of LBs in both DLB and AD. Increased neocortical expression of α-synuclein mRNA is found in DLB, but not in LBV or AD[48], in contrast to the up-regulation of this protein in the same region in preclinical stages of AD dementia[31]. Loss of soluble α-synuclein has been demonstrated semi-quantitatively in LBV in the medial temporal lobe[49]. This finding is similar to that from our study. Interestingly, the medial temporal lobe expression of α-synuclein in AD is similar to that of the control group, despite AD and LBV having a similar extent of AD pathology. This may be due to the presence of transient α-synuclein intraneuronal inclusions in the medial temporal lobe, characteristic for AD, but not DLB[50]. Similarly, we cannot exclude the possibility of a transient medial temporal lobe up-regulation of α-synuclein in AD, similar to that described for neocortex[31]. This will need to be confirmed in further, more extensive studies.

The loss of α-synuclein in the medial temporal lobe of LBV cannot be explained solely on the basis of its incorporation into the dystrophic neurites within the plaque corona, as described recently[51]. Since a similar level of neuritic plaques is also found in AD (Mukaetova-Ladinska et al., unpublished data), this suggests that loss of this protein may be regulated via another mechanism, either by its incorporation into LBs or by its own neurotoxicity[52,53]. None of the subjects in the LBV group had LBs in their medial temporal lobes; a small number were found in the brainstem and midbrain, and only a few in the neocortical areas of the frontal and temporal neocortices. This suggests that if the depletion of α-synuclein is due to the presence of LBs, it will be due largely to the altered connectivity between the medial temporal lobe and the subcortex.

The relationship between LB formation and AD-related pathology remains unclear. The incidence of LBs increases with the progression of AD[2], suggesting that the neurodegenerative changes of AD may contribute to LB formation, either via altered metabolism of the amyloid precursor protein or through the post-translational modification and/or truncation of tau protein. Our study indicates that the connection between these two clinical and neuropathological entities may be more complex, with the differences occurring at an early stage, and being restricted to the altered synaptic and cytoskeletal processing that precedes the more advanced stages of AD. It is also possible that altered metabolism of α-synuclein, its aggregation and subsequent loss of soluble α-synuclein, may affect other synaptic proteins involved in the docking and fusion of synaptic vesicles. This could explain not only the subtle clinical differences between LBV and AD, but also the differences in their cholinergic profiles[54], with LBV having more profound loss of ChAT even in the earliest stages of illness[55]. At this point, we can only speculate about the molecular mechanisms underlying the synaptic loss seen in LBV, and how the latter is related to the distinct clinical phenotypes in different dementia syndromes. Further correlative cliniconeuropathological and biochemical studies are now needed to address this.

We recently described a selective reduction in the t-SNARE complex in schizophrenia[40] accompanied by depletion of MAP2 in the hippocampus (Mukaetova-Ladinska et al., unpublished), similar to that described for LBV in the present study. Since LBs are not a characteristic of the elderly schizophrenic brain, it is unlikely that the selective t-SNARE complex is linked to the formation of these intraneuronal inclusions. We are, therefore, left with the hypothesis that this loss may be characteristic of the clinical syndrome shared between the two clinical entities, rather than being intrinsic to the formation of LBs in AD.

One of the limitations of the current study is the small number of LBV analyzed cases. The results from the current study, therefore, represent pilot findings, and they need to be explored further on larger numbers of not only LBV individuals, but also subjects with DLB, in order to address in detail the development of synaptic loss that occurs during the disease progression, and to determine to what extent this is due to the formation of LBs and/or associated neurodegenerative pathologies. The role of the dual Alzheimer and LB pathology in LBV needs to be explored in further brain areas in order to determine their impact on different neurotransmitter systems involved in the disease process. This may result in new therapeutic approaches for this group of patients, where cholinergic and dopaminergic treatments are solely symptomatic, and not always beneficial.

## ACKNOWLEDGMENTS

We are indebted to all the patients and their families who participated in the study. Brain tissue came from the collection of the Cambridge Brain Bank. We thank Mrs. A. O'Sullivan for liaison with the patients and their families and Mrs. Caroline Kirk and Mrs. Alyson Goldwater for secretarial support. This study has been generously supported by an MRC Grant U.K. (EBM-L, JH, CMW, CRH, JX), a Canadian Institutes of Health Research grant (WGH), CONACyT Grant, Mexico and Alzheimer's Research Fund, Cambridge (FG-S). The clinical and epidemiological study was supported by MRC, Grant, U.K., RIA Grant, U.K., and Ed Stanley Foundation, U.K.
